# Supplementary material for: Learning curve and surgical outcome of robotic assisted colorectal surgery with ERAS program
Source: Sci Rep. 2022 Nov 29;12:20566. doi: 10.1038/s41598-022-24665-w (PMC9709162; doi:10.1038/s41598-022-24665-w)
Supplement: Supplementary file 2 — Supplementary Information 2. [file 41598_2022_24665_MOESM2_ESM.zip › RAL Raw data-Ñ[▒K/055/HS_ñw╝╨░O▒Kñσ.pdf]

正式摘要(電子簽章：000000209200861)		
W95 / W95 ☐ 英文標題(注意:無英文姓名-請補病人基本檔!)	☐ 【讀取】憑證	

臺中榮民總醫院
出院病歷摘要

轉入號 : 轉出號 :

[redacted]

**(8)**流水編號	
**(9)**入院日期	110/01/22 (2021) 直腸外科 W95 - 39
**(10)**轉科日期	
**(11)**出院日期	110/01/28 (2021) 住院天數計 6 日

診斷

- 入院 :rectal cancer
  Hypertension
  BPH
  gout
- 出院 :rectal cancer s/p robot assisted LAR on 2021/1/23

  Hypertension
  BPH
  gout

主訴

- bloody stool about 1 year

病史

- This 71 years old male patient has past history of hypertension, gout, BPH, left basal ganglion ICH. He recieved laparoscopic cholecystecomy on 2017/11. He complained bloody stool for about 1 year and he came to Puli. branch for help on 2020/12. Colonoscopy showed rectal tumor, 5cm from anal verge. Biopsy report showed adenocarcinoma. He was transferred to our hospital. PET showed no obvious distal metastasis. Short course radiotherapy was started on 2021/1/15. This time, he was admitted for surgical treatment.

體檢發現

- PS 70%
  - General appearance :
    - easy looking , conscious clear , without cardiopulmonary distress
    - Vital signs自動帶入date 體溫(BT) 脈搏(P) 呼吸(R) 血壓(BP) 身高(BH) 體重(BW) BMI
      20210122 36℃ 81次/分 17次/分 136/80mmHg 160.6cm 64.7kg 25.1
  - HEENT :
    - Eyes: no anemic conjunctiva, no icteric sclera, normal EOM
    - ENT: no otorrhea, no rhinorrhea, no erythematous mucosa, no injected tonsil, no deformity
  - Neck: supple, freely movable, trachea midline,no deformity, no engorged jugular vein , no palpable thyroid or lymph node enlargement
  - Chest:
    - Inspection: Grossly normal, symmetric and fully expansion, no accessory muscle used, no operation scar, no spider angiomata, no gynecomastia
    - Palpation: no palpable mass or nodule, no subcutaneous emphysema, no tenderness
    - Ausculation: clear breathing sound
  - Heart:
    - Percussion: normal heart size
    - Ausculation: regular heart beat, normal S1, S2, no gallop, no murmur
  - Abdomen:

-
  - Inspection: in
    - flat contour, symetrically, no scar, no stria, no dilated veins, no rashes
  - Ausculation: normoactive bowel sound, no abdominal bruits
  - palpation: soft, no muscle guarding or rigidity, no local tenderness, no rebounding pain, no palpable mass, no palpable pulsation
  - Percussion: no shifting dulllness, no hepatomegaly, no splenomagaly
-

Peripheral pulsation :	CA	BA	RA	FA	PA	PTA	DPA
Right side :	++	++	++	++	++	++	++/+++
Left side :	++	++	++	++	++	++	++/+++

- Genito-anus: no tenderness or palpable mass lesion or rectal shelf, normal external genitalia, normal anal tone, brown stool

CRS CONDITION

- Rectodigital examination: -
- CEA: 27.61 ng/ml
-
  - Lung

手術日期及方法

- 手術日期及方法20210123 1. Robotic (XI) assisted TME+ loop ileostomy
  2. Intraoperative colonoscopy : (-).
  3. High ligation (+) : At IMA root and IMV (Via robotic hemolok).
  4. Take down from the splenic flexure : (-)
  5. Robotic EndoGIA (45-4.3) X2 + EEA (29mm) + OpenGIA (80-3.8) X1 were applied to complete the side-to-end anastomosis
  6. Proximal cut end: 19 cm, distal cut end: 1.5 cm
  7. No drainage tube was inserted
  8. ADHESION BARRIER FILM was placed
  9. Loop ileostomy (About 15cm from IC valve) was done over RLQ of abdomen

住院治療經過

- After admission, operation with robot assisted LAR was done on 1/23 as scheduled. We let him try water and diet as ERAS protocol. We prescribed loperamide due to much ostomy output. Rod was removed on POD5. Under the stable condition, he was discharged on 1/28.

合併症

- nil

一般檢查

- 累積報告 :

DATE	TIME	ALB	TP	BIL.T	BIL.D	ALKP	AST	ALT	LDH	CHOL	TG	HDL-C	LDL	UA	NH3	AMY
1100123	1809	3.5		1.11			45	46								
1100128	0902	3.5		0.8				67								

DATE	TIME	WBC	RBC	HGB	HCT	MCV	PLT	BLST	NEUT	BAND	SEG	LYM	MON	EOS	BAS
1100123	1809	5920		11.7			85		86.0			9.5	3.9	0.3	0.3
1100128	0931	4970	4.18	12.5			84		62.6			20.1	9.5	7.2	0.6

DATE	TIME	NA	K	CL	CA	BUN	CREAT	Mg	P	CRP	ESR	PCT	LIPASE	LACTATE
1100123	1809	142	2.5			20	1.54	1.5						
1100124	0857		2.6											
1100128	0902	139	2.9	97	8.7	16	1.42							

特殊檢查

病理報告

其它

出院時情況

- 3.改門診治療

出院指示

-

藥名	劑量	劑量單位	用法	頻次	數量	數量單位	現況	天數	單位
Ultracet tab	1	TAB	PO	BIDPRN	12	TAB	TH	6	天
Loperamide cap 2mg	2	CAP	PO	QD	12	CAP	TH	6	天
$Biofermin tab	2	TAB	PO	TID	36	TAB	TH	6	天
Radi-K tab 595 mg	1	TAB	PO	QD	6	TAB	TH	6	天

出院計劃

主治醫師_2062F 蔣鋒帆 / 住院醫師_2331F 陳凱祥

正式摘要**(**電子簽章：**000000209200861)**

W95 / W95

doctorRank:B
